# Regulatory SVA retrotransposons and classical HLA genotyped-transcripts associated with Parkinson’s disease

**DOI:** 10.3389/fimmu.2024.1349030

**Published:** 2024-03-25

**Authors:** Jerzy K. Kulski, Shingo Suzuki, Takashi Shiina, Abigail L. Pfaff, Sulev Kõks

**Affiliations:** ^1^ Department of Molecular Life Science, Tokai University School of Medicine, Isehara, Kanagawa, Japan; ^2^ Health and Medical Science, Division of Immunology and Microbiology, School of Biomedical Sciences, The University of Western Australia, Nedlands, WA, Australia; ^3^ Perron Institute for Neurological and Translational Science, Perth, WA, Australia; ^4^ Centre for Molecular Medicine and Innovative Therapeutics, Murdoch University, Perth, WA, Australia

**Keywords:** major histocompatibility complex (MHC), human leucocyte antigen (HLA), SINE-VNTR-Alu (SVA), expression quantitative trait loci (eQTL), Parkinson’s disease (PD), Parkinson’s progression markers initiative (PPMI)

## Abstract

**Introduction:**

Parkinson’s disease (PD) is a neurodegenerative and polygenic disorder characterised by the progressive loss of neural dopamine and onset of movement disorders. We previously described eight SINE-VNTR-Alu (SVA) retrotransposon-insertion-polymorphisms (RIPs) located and expressed within the Human Leucocyte Antigen (HLA) genomic region of chromosome 6 that modulate the differential co-expression of 71 different genes including the HLA classical class I and class II genes in a Parkinson’s Progression Markers Initiative (PPMI) cohort.

**Aims and methods:**

In the present study, we (1) reanalysed the PPMI genomic and transcriptomic sequencing data obtained from whole blood of 1521 individuals (867 cases and 654 controls) to infer the genotypes of the transcripts expressed by eight classical HLA class I and class II genes as well as *DRA* and the *DRB3/4/5* haplotypes, and (2) examined the statistical differences between three different PD subgroups (cases) and healthy controls (HC) for the HLA and SVA transcribed genotypes and inferred haplotypes.

**Results:**

Significant differences for 57 expressed HLA alleles (21 HLA class I and 36 HLA class II alleles) up to the three-field resolution and four of eight expressed SVA were detected at *p<0.05* by the Fisher’s exact test within one or other of three different PD subgroups (750 individuals with PD, 57 prodromes, 60 individuals who had scans without evidence of dopamine deficits [SWEDD]), when compared against a group of 654 HCs within the PPMI cohort and when not corrected by the Bonferroni test for multiple comparisons. Fourteen of 20 significant alleles were unique to the PD-HC comparison, whereas 31 of the 57 alleles overlapped between two or more different subgroup comparisons. Only the expressed HLA-DRA*01:01:01 and -DQA1*03:01:01 protective alleles (PD v HC), the -DQA1*03:03:01 risk (HC v Prodrome) or protective allele (PD v Prodrome), the -DRA*01:01:02 and -DRB4*01:03:02 risk alleles (SWEDD v HC), and the *NR_SVA_381* present genotype (PD v HC) at a 5% homozygous insertion frequency near *HLA-DPA1*, were significant (*Pc<0.1*) after Bonferroni corrections. The homologous *NR_SVA_381* insertion significantly decreased the transcription levels of *HLA-DPA1* and *HLA-DPB1* in the PPMI cohort and its presence as a homozygous genotype is a risk factor (*Pc=0.012*) for PD. The most frequent *NR_SVA_381* insertion haplotype in the PPMI cohort was NR_SVA_381/DPA1*02/DPB1*01 (3.7%). Although HLA C*07/B*07/DRB5*01/DRB1*15/DQB1*06 was the most frequent HLA 5-loci phased-haplotype (n, 76) in the PPMI cohort, the *NR_SVA_381* insertion was present in only six of them (8%).

**Conclusions:**

These data suggest that expressed SVA and HLA gene alleles in circulating white blood cells are coordinated differentially in the regulation of immune responses and the long-term onset and progression of PD, the mechanisms of which have yet to be elucidated.

## 1 Introduction

Parkinson’s disease (PD), familial and sporadic, is the second most common human neurodegenerative disease after Alzheimer’s disease with almost 90,000 people in the USA diagnosed each year, and a 2019 world-wide prevalence rate of 8.5 million individuals that is increasing (1). PD pathology is age-related and characterised by progressive degeneration of dopaminergic neurons in the *substantia nigra* and other brainstem nuclei, with accumulation of tau and alpha-synuclein deposits (Lewy body inclusions) throughout the peripheral and central nervous systems (2–5). Essential differential observations accompanying PD subtypes include loss of dopamine, bradykinesia (movement disorders), rigidity, tremor and a range of non-motor symptoms such as cognitive impairment and sleep disturbance (6, 7). The primary and secondary causes of PD may involve genetic, environmental, metabolic and immunological factors with various non-neurological features and varying overlap with age-related autoimmune diseases such as multiple sclerosis, amyotrophic lateral sclerosis, thyroid diseases and rheumatoid myalgia (3, 8–11). In regard to the effect of the environment and immunogenetics, Braak et al. (12) postulated that an unknown viral or bacterial infection in the neurons of the gut and/or nasal cavity initiated the onset of sporadic PD with specific alpha-synuclein spreading and eventual Lewy body formation and glial neuroinflammatory activation. Considerable preclinical, clinical and laboratory evidence supports Braak’s hypothesis of PD progression, although the specific mechanisms, stages and pathways still have to be elucidated (13, 14). Recent animal *in vitro* studies and human neuropathological examinations suggest that neuronal antigen presentation may have a role in PD and other neurodegenerative disorders (15).

Although the aetiology of sporadic PD remains unknown, the immune system has an important role in this disease (3, 8–11). The protective effect of nonsteroidal anti-inflammatory drugs in animal models and epidemiological studies underscores the role of neuroinflammation in PD (16). Large numbers of microglia expressing human leucocyte antigen (HLA)-DR have been detected in the brain of PD patients, particularly in areas of maximal neurodegeneration (15, 17). *Leucine-rich repeat kinase 2* (*LRRK2*), a risk gene of PD, is highly expressed in microglia, monocytes and other immune cells (18), and has been reported to be associated with an increasing risk of Crohn’s disease, an inflammatory bowel disease and other autoimmune diseases (19–21). Alpha-synuclein specific T cell reactivity is associated with HLA-DRB1*15:01 and -DRB5*01:01 (22, 23), and with preclinical and early PD (24, 25), and the infiltration of CD4+ lymphocytes into the brain contributes to neurodegeneration in a mouse model of PD (15, 26). At least 90 genetic loci have been associated with PD risk in genome-wide association studies (GWAS), including the *HLA-DRA*, -*DRB*, and *-DQ* genes within the Major Histocompatibility Complex (MHC) class II region on the short arm of chromosome 6 at 6p21.3 (27, 28).

HLA class I and class II molecules are polymorphic cell-membrane-bound glycoproteins that present antigens to circulating CD8+ and CD4+ T-lymphocytes, and regulate the innate and adaptive immune responses including autoimmunity, infectious diseases and transplantation outcomes (29–31). The MHC or HLA genomic region encodes at least 160 genes within ~ 3 to 4 MB including three distinct structural regions designated as class I, class II and class III. Of the 32 HLA genes, the classical HLA class I genes, *HLA-A*, *-B* and *-C*, and the classical HLA class II genes, *HLA-DR, -DQ* and *-DP*, are characterised by an extraordinary large number of polymorphisms, whereas the non-classical HLA class I genes, such as *HLA-E, -F* and *-G*, are differentiated by their tissue-specific expression and limited polymorphism (32, 33). Several GWASs have shown an association between the HLA locus and the risk of PD especially involving the HLA class II gene SNPs of *HLA-DQA1, -DQA2, -DQB1, -DRB1*, and *-DRB5* (27, 34–36).

Most studies of PD association with HLA class I and class II alleles are limited in scope and power mainly because of small sample numbers and limited resolution of HLA typing methods. Studies with more than 500 PD cases suggest that HLA genes have a role in risk or protection in PD progression. The study by Saiki et al. (37) of a UK study group (528 PD cases and 3430 controls) revealed that HLA-DRB1*03 and -DQB1*05 allele groups were possible PD risk alleles whereas HLA-DRB1*04 and -DQB1*03 might be protective. Wissemann et al. (35) in an analysis of 2843 European PD cases from two separate cohorts including healthy controls found that the HLA class II risk alleles were HLA-DRB1*15:01, -DQA1*01:02 and -DQB1*06:02, and the protective alleles were -DRB1*04:04, -DQA1*03:01, and -DQA1*03:02. They also suggested that HLA-B*07:02 and -C*07:02 are part of an HLA risk haplotype, whereas HLA-B*40:01 and -C*03:04 are protective alleles. Hollenbach et al. (38) in a sequencing and typing analysis of 11 classical HLA loci using 1597 PD and 1606 controls found strong protective effects of HLA-DRB1*04:01 and HLA-DQB1*03:02, but no significant differences between cases and controls for alleles of any class I locus (*HLA-A*, *-B*, and *-C*) or class II loci *HLA-DPA1, -DPB1, -DRB3, -DRB4*, and *-DRB5*. They also proposed that HLA susceptibility to PD can be explained by a specific combination of amino acids at positions 70–74 on the HLA-DRB1 molecule referred to as the ‘shared epitope’ (SE) and that the SE in combination with valine at position 11 (11-V) is highly protective in PD, but a risk with the absence of 11-V. More recently, Yu et al. (34) used 13,770 European PD patients in a meta-analysis of multiple cohorts from eight independent sources to confirm that HLA-DRB1*04:01, -DRB1*04:04, -DQA1*03:01 and -DQB1*03:02 were protective. They concluded that the effect of the *HLA-DRB1* gene in susceptibility for PD is small and does not merit routine HLA typing in PD. An earlier study of Chinese Han (567 PD cases and 746 controls) indicated that HLA-DRB1*03:01 was a risk allele, whereas HLA-DRB1*04:06 was a protective allele in their study of only *HLA-DRB1* alleles (39). More studies of the association between HLA genotypes and PD are needed to understand the role of HLA in the disease processes of PD and how HLA genes and alleles might be interlinked with accompanying autoimmune diseases, especially those that show non-neurological symptoms associated with PD such as sleep disorder and a decrease in *HLA-DR* expression (40).

Apart from protein coding genes, numerous repeat elements (REs) within the human genome have been associated with PD including *SINE-R-VNTR-Alu* (SVA) retrotransposon insertion polymorphisms (RIPs), such as a SVA that is inserted in the *TAF1* gene that has been associated with the disease X-linked dystonia-parkinsonism, and at least five other SVA inserted within the PD (*PARK*) gene loci of different chromosomes (41, 42). Recently, expression quantitative trait loci (eQTL) of different SVAs and their effect on the regulation of gene expression were identified and described for a Parkinson’s Progression Markers Initiative (PPMI) cohort using whole genome sequence and transcriptome data obtained from the blood of more than a thousand individuals (43). Also, there are SVAs within the MHC genomic region that are expressed and can regulate the expression of HLA genes (44). At least eighteen SVA polymorphic insertions were mapped previously within the MHC class I, II and III regions, and some were found to be haplotypic or haplospecific for particular HLA gene alleles that varied in frequency between European, Japanese and African American populations (45). For example, the *SVA-HF*, *SVA-HA*, and *SVA-HC* were inserted at a relatively low frequency (<0.2) in European populations and strongly associated with the HLA 7.1 ancestral haplotype, but not with the 8.1 haplotype (46, 47).

A PPMI clinical protocol was established in 2010 to acquire comprehensive longitudinal within-participant clinical, imaging, genomic, transcriptomic and biomarker data for three main cohorts, (1) PD with and without genetic risk variants, (2) prodromes (nonmotor features) at risk of PD, and (3) healthy controls with no neurological disorder and no first degree relative, currently aimed at enrolling 4000 participants at about 50 sites worldwide (48). We associated the regulatory properties of 8 SVA RIPs located within the class I and class II MHC regions of the PPMI cohort with the differential co-expression of 71 genes within and 75 genes outside of the MHC region, including all the classical class I and class II genes (44). A limitation of this SVA-HLA eQTL study was the absence of HLA allelic data to associate with the SVA genotypes and for stratifying the statistical differences between PD, prodromes and healthy controls within the PPMI cohort.

**Figure 1:**
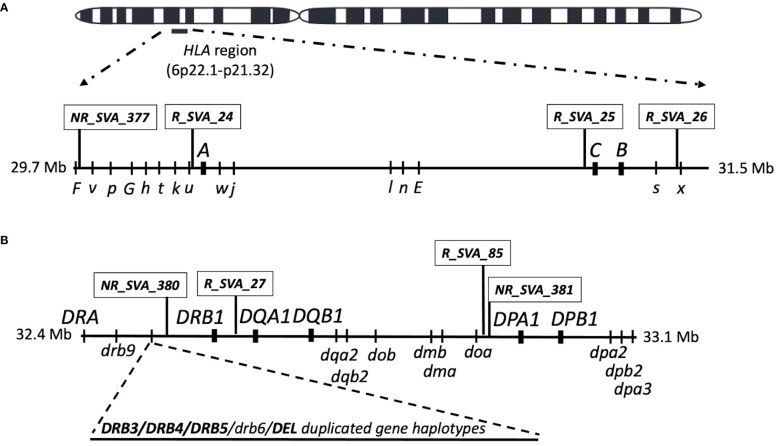
Location map of the SVA and classical HLA class I and class II genes on chromosome 6 that were transcribed in blood cells in this study. **(A)** is the HLA class I region showing the relative SVA and the three classical class I HLA gene loci above the horizontal line investigated in this study. The relative position of HLA nonclassical gene (capital letter) and pseudogene (lower case letters) loci are shown below the horizontal line. **(B)** is the classical class II region showing the relative SVA and five classical class II HLA gene loci above the horizontal line investigated in this study. The relative position of the HLA nonclassical class II genes and pseudogene loci are shown below the horizontal line. The region between *HLA-DRB9* and -*DRB1* that harbours the structural variants for the *HLA-DRB3*, *-DRB4, -DRB5*, and *-DRB6* genes and deletion are indicated by the dashed lines as an added horizontal extension. The Class III region that is located between the class I and class II regions is not shown in the figure. The location of all genes, pseudogenes and SVA are not shown to exact genomic scale.

The purpose of our current study was to undertake an analysis of the expression of ten classical class I (*HLA-A, -B, -C*) and class II (*HLA*-*DRA, -DRB3/4/5 -DRB1, -DQB1, -DQA1, -DPA1, -DPB1*) gene alleles in the context of the eight regulatory SVA RIPs expressed within the MHC genomic region (**Figure 1**) that we had previously studied (44). The main aims of this study were to determine:

(a) the prevalence of the expressed HLA classical alleles and inferred haplotypes for the entire PPMI cohort, cases and controls.
(b) the HLA allelic and haplotypic statistical differences between PD, healthy controls (HC), prodromal PD and scans without evidence of dopamine deficits (SWEDD).
(c) the inferred SVA haplotypes and their association with HLA gene alleles.
(d) statistical differences between HLA & SVA alleles and PD, HC, prodromes and SWEDD.

Our RNA data analysis confirms that SVAs are eQTLs for classical HLA class I and class II alleles, and suggests that coordinated SVA and HLA gene expression might influence PD onset or progression via the adaptive immune system.

## 2 Materials and methods

### 2.1 Parkinson’s progression markers initiative datasets

The PPMI and database is an ongoing longitudinal, observational, multicentre study of PD with an overall goal to identify biological and genetic markers of disease progression, accelerate therapeutic trials and reduce progression of PD disability (48). The PPMI cohort data were downloaded from http://www.ppmi-info.org/data (accessed on 19 January 2021) as previously described by Koks et al. (41). Transcriptome (RNAseq) data obtained from whole blood samples together with genetic and clinical data of 1521 individuals reported as mostly white Americans within the PPMI cohort consist of four subgroups, (1) 750 individuals with Parkinson disease (PD), (2) 57 with prodromal PD (Prodrome), (3) 60 individuals who had scans without evidence of dopamine deficits (SWEDD), and (4) 654 healthy controls (HC). The entire PPMI cohort with the four subgroups were analysed to determine the association between the transcribed SVA and classical HLA genes, each at eight loci, within the class I and class II regions of the human MHC (**Figure 1**).

### 2.2 SVA and HLA genotypes

Regulatory effects of SVA on HLA transcription levels were inferred statistically by eQTL analysis using the Matrix eQTL software (49) and described previously (44). Fastq files of whole-blood RNAseq were downloaded from the PPMI database and the referenced SVA (R_SVA) and non-referenced SVA (NR_SVA) (**Figure 1**) were located, genotyped and identified within or outside the MHC genomic region with the assistance of the software tools, *Delly2* structural variant caller and the transcript counters *Salmon* and *DESeq2*, as previously described (41, 44). All the transcripts’ of 1521 individuals downloaded as PPMI blood RNAseq.bam files were used to identify the genotypes of ten classical class I and class II HLA genes using the *arcasHLA* software tool described by Orenbach et al. (50). *DRB3, DRB4* and *DRB5* were counted as a single locus or gene, including the designated ‘*DRB3DRB4DRB5 absent*’, which is the haplotype with no *DRB3, DRB4, DRB5* locus. The HLA transcripts were ‘genotyped’ at least to the three-field resolution (eg., A*02:01:01) whereby the first field represents the ancestral allele group (e.g., A*02), the second field represents protein type and the third field represents synonymous changes in coding regions.

### 2.3 Statistical analysis

The p-values, odds ratios (OR), and 95% confidence intervals (CI) were calculated using Fisher’s exact test using R software (R version 4.1.3). For multiple testing, the Bonferroni correction was applied, and the observed p-values were adjusted by multiplying them by the number of alleles at each HLA locus to obtain *Pc* values (Bonferroni-corrected *Pc*-values). The estimation of haplotypes was performed using the PHASE program v2.1.1 (51) and are referred to in this study as phased-haplotypes.

## 3 Results

### 3.1 Common medical disorders associated between PD and HC

The aetiology of PD appears to be multifactorial involving aging, genetics, environmental factors (9), reflected by other inflammation-related disorders or autoimmune diseases (20, 52, 53). **Table 1** shows a list of common diseases or disorders in 318 PD patients and 264 healthy controls (free of PD) in a subset of the PPMI cohort. The most significant risk factors (Pc<0.1) associated with PD in this subset of PD patients (average age of 61 years) is scoliosis (n, 9 v 0), and sleep disturbances (n, 72 v 33). Thyroid disease including hyperthyroidism is a risk factor in 75 of the PD patients by the Fisher’s exact test with a *p*-value of 0.012.

**Table 1:** Common medical diagnoses in 318 PD patients and 264 healthy controls (free of PD).

Common medical diagnoses	n		%		OR	95% CI				
Available medical reports	PD	HC	PD	HC		Lower	Upper	P-value	Pc-value	PD risk
allergy	6	68	1.9%	25.8%	0.06	0.02	0.13	< 2.20E-16	<7.26E-15	protective
arthritis	58	81	18.2%	30.7%	0.50	0.34	0.76	0.00060	0.01992	protective
asthma	12	14	3.8%	5.3%	0.70	0.29	1.67	0.42320	1	
atrial fibrillation	14	9	4.4%	3.4%	1.30	0.52	3.48	0.67030	1	
basal cell carcinoma	12	12	3.8%	4.5%	0.82	0.33	2.04	0.67940	1	
breast cancer	2	6	0.6%	2.3%	0.27	0.03	1.54	0.14980	1	
coronary artery disease	11	6	3.5%	2.3%	1.54	0.51	5.14	0.46500	1	
dermatitis	8	4	2.5%	1.5%	1.68	0.44	7.69	0.56050	1	
diabetes	29	34	9.1%	12.9%	0.68	0.39	1.19	0.17990	1	
Erectile Dysfunction	25	8	7.9%	3.0%	2.73	1.17	7.12	0.01197	0.39501	risk
fibromyalgia	3	6	0.9%	2.3%	0.41	0.07	1.94	0.31210	1	
genital herpes	5	3	1.6%	1.1%	1.39	0.27	9.03	0.73410	1	
gout	5	0	1.6%	0%	Inf	0.76	Inf	0.06681	1	
hypercholesterolemia	58	44	18.2%	16.7%	1.12	0.71	1.76	0.66200	1	
hyperlipidemia	45	57	14.2%	21.6%	0.60	0.38	0.94	0.02142	0.70686	protective
hypertension	109	88	34.3%	33.3%	1.04	0.73	1.50	0.86040	1	association
melanoma	8	5	2.5%	1.9%	1.34	0.38	5.26	0.78020	1	
Myasthenia Gravis	3	0	0.9%	0%	Inf	0.34	Inf	0.25520	1	
neck disorder or pain	5	13	1.6%	4.9%	0.31	0.09	0.94	0.02835	0.93555	protective
neuropathy	21	14	6.6%	5.3%	1.26	0.60	2.74	0.60050	1	
osteoporosis	26	23	8.2%	8.7%	0.93	0.50	1.76	0.88120	1	
prostate carcinoma	13	9	4.1%	3.4%	1.21	0.47	3.26	0.82790	1	
psoriasis	10	14	3.1%	5.3%	0.58	0.23	1.43	0.21350	1	
restless leg syndrome	8	9	2.5%	3.4%	0.73	0.24	2.17	0.62350	1	
scoliosis	9	0	2.8%	0%	Inf	1.67	Inf	0.00487	0.16084	risk
shingles herpes	1	3	0.3%	1.1%	0.28	0.01	3.45	0.33390	1	
sleep disturbances, insomnia, sleep apnea	72	33	22.6%	12.5%	2.05	1.28	3.32	0.00164	0.05415	risk
squamous cell carcinoma	4	0	1.3%	0%	Inf	0.55	Inf	0.13020	1	
thyroid disease and hyperthyroidism	75	40	23.6%	15.2%	1.73	1.11	2.72	0.01205	0.39765	risk
urinary tract infection	8	8	2.5%	3.0%	0.83	0.27	2.56	0.80110	1	

665 listed diseases in 262 individuals (82.4%) with PD (average age of 61).

611 listed diseases 234 individuals (88.6%) without PD (average age of 60).

‘Inf’ is an infinite value due to a zero value in HC.

### 3.2 HLA genotyped transcripts and statistical associations within case-control comparisons

The HLA genotypes of ten classical class I and class II genes of 1521 individuals within the PPMI cohort inferred from the transcription data are presented in **Supplementary Table 1**, and their overall frequencies are shown in **Supplementary Table 2**. The top six *HLA-A, -B, -C, -DRB1, -DQB1*, -*DQA1*, -*DPA1, -DPB1* allele frequencies in the PPMI cohort are shown in **Table 2**, confirming that the PPMI cohort consists mostly of white European or North American ancestry (33).

**Table 2:** Top six inferred allele frequencies for HLA-A, -B, -C, -DRB1, DQA1, -DQB1, -DPA1 and -DPB1 transcripts.

Locus	Allele name	Number of genotypes				
		PD	PRODROME	SWEDD	HC	% Total
HLA-A	A*02:01:01	308	35	28	250	20.41
HLA-A	A*01:01:01	237	13	19	183	14.86
HLA-A	A*03:01:01	170	11	18	139	11.11
HLA-A	A*24:02:01	119	9	12	136	9.07
HLA-A	A*26:01:01	80	7	4	93	6.05
HLA-A	A*11:01:01	64	4	2	55	4.11
HLA-B	B*08:01:01	136	8	15	100	8.51
HLA-B	B*07:02:01	108	5	17	97	7.46
HLA-B	B*38:01:01	81	8	5	102	6.44
HLA-B	B*14:02:01	94	1	2	96	6.34
HLA-B	B*44:02:01	90	11	10	78	6.21
HLA-B	B*35:01:01	90	5	6	69	5.59
HLA-C	C*04:01:01	204	12	10	190	13.68
HLA-C	C*07:01:01	203	14	20	140	12.39
HLA-C	C*06:02:01	147	12	7	134	9.86
HLA-C	C*12:03:01	130	9	8	134	9.24
HLA-C	C*07:02:01	130	7	19	108	8.68
HLA-C	C*08:02:01	100	3	2	98	6.67
HLA-DRB1	DRB1*07:01:01	207	14	16	192	14.10
HLA-DRB1	DRB1*03:01:01	145	13	15	107	9.20
HLA-DRB1	DRB1*15:01:01	131	11	14	91	8.12
HLA-DRB1	DRB1*11:04:01	118	6	5	108	7.79
HLA-DRB1	DRB1*01:01:01	100	7	6	90	6.67
HLA-DRB1	DRB1*13:01:01	80	9	3	78	5.59
HLA-DQA1	DQA1*05:05:01	270	12	13	208	16.54
HLA-DQA1	DQA1*02:01:01	206	14	16	193	14.10
HLA-DQA1	DQA1*01:02:01	197	18	22	140	12.39
HLA-DQA1	DQA1*03:01:01	128	6	18	157	10.16
HLA-DQA1	DQA1*05:01:01	148	11	16	111	9.40
HLA-DQA1	DQA1*01:03:01	110	10	4	116	7.89
HLA-DQB1	DQB1*03:01:01	259	18	19	207	16.54
HLA-DQB1	DQB1*05:01:01	191	15	10	195	13.51
HLA-DQB1	DQB1*02:02:01	148	8	8	151	10.36
HLA-DQB1	DQB1*06:02:01	145	11	18	110	9.34
HLA-DQB1	DQB1*03:02:01	117	7	18	137	9.17
HLA-DQB1	DQB1*02:01:01	138	13	15	102	8.81
HLA-DPA1	DPA1*01:03:01	1151	83	92	1047	78.01
HLA-DPA1	DPA1*02:01:01	215	18	12	174	13.77
HLA-DPA1	DPA1*02:01:02	48	1	7	28	2.76
HLA-DPA1	DPA1*02:02:02	34	3	4	15	1.84
HLA-DPA1	DPA1*02:07:01	16	0	2	12	0.99
HLA-DPA1	DPA1*01:04:01	9	2	2	16	0.95
HLA-DPB1	DPB1*04:01:01	577	42	46	545	39.78
HLA-DPB1	DPB1*02:01:02	225	14	13	226	15.71
HLA-DPB1	DPB1*04:02:01	182	6	12	135	11.01
HLA-DPB1	DPB1*03:01:01	89	9	11	66	5.75
HLA-DPB1	DPB1*01:01:01	69	5	8	42	4.08
HLA-DPB1	DPB1*104:01:01	46	3	2	46	3.19
total number of genotypes		1500	114	120	1308	3042

The significant differences at *p<0.05* detected by the Fisher’s exact test for 20 different HLA alleles (7 HLA class I and 13 HLA class II alleles) up to the three-field resolution in the statistical comparison between HC (n, 654) and PD (n, 750) are shown in **Table 3**. There are 8 risk alleles (3 HLA class I and 5 HLA class II alleles) and 12 protective alleles (4 HLA class I and 8 class II alleles) within this comparison. The protective alleles, HLA-DRA*01:01:01 and -DQA1*03:01:01, are the only two significant (*Pc<0.1*) alleles after Bonferroni correction for multiple testing. Some notable allelic differences between PD and HC at the *p<0.05* level are HLA-B*40:02:01, -DRB1*11:02:01 and -DPA1*02:02:02 with relatively high *OR* levels (3.68, 4.38 and 2, respectively) and -DRB1*04:02:01, -DRB1*04:04:01, -DQA1*03:01:01, -DQB1*03:02:01, -DPA1*01:03:01 and -DPB1*16:01:01 with relatively low *OR* levels (0.64, 0.56, 0.68, 0.72, 0.82, and 0.17).

**Table 3:** Significantly expressed HLA genotypes in healthy controls (HC) versus Parkinson Disease (PD).

HC, n=654 v PD, n=750												
20 alleles	n		%		PD	HC	OR	95% CI		P < 0.05	P< 0.1	Risk or
Allele name	PD	HC	PD	HC	Other	Other		Lower	Upper	P-value	Pc-value	protective
A*31:01:02	32	14	2.13%	1.07%	1468	1294	2.01	1.04	4.11	0.035850	1	risk
B*40:02:01	25	6	1.67%	0.46%	1475	1302	3.68	1.47	10.99	0.001917	0.151443	risk
C*07:01:01	203	140	13.53%	10.70%	1297	1168	1.31	1.03	1.66	0.024200	1	risk
DRB5*01:01:01	136	90	9.1%	7.0%	1352	1204	1.35	1.01	1.80	0.03693	0.59088	risk
DRB1*11:02:01	10	2	0.67%	0.15%	1490	1306	4.38	0.93	41.21	0.043470	1	risk
DQB1*05:03:01	53	27	3.53%	2.06%	1447	1281	1.74	1.07	2.89	0.022560	0.879840	risk
DPA1*02:02:02	34	15	2.27%	1.15%	1466	1293	2.00	1.05	3.97	0.02939	0.529020	risk
DPB1*13:01:01	47	24	3.13%	1.83%	1453	1284	1.73	1.03	2.98	0.030210	1	risk
A*24:02:01	119	136	7.93%	10.40%	1381	1172	0.74	0.57	0.97	0.025160	1	protective
B*15:03:01	0	4	0%	0.31%	1500	1304	0	0	1.32	0.046970	1	protective
B*38:01:01	81	102	5.40%	7.80%	1419	1206	0.68	0.49	0.92	0.011340	0.895860	protective
C*02:10:01	0	4	0%	0.31%	1500	1304	0	0	1.32	0.046970	1	protective
DRA*01:01:01	949	889	63.3%	68.0%	551	419	0.81	0.69	0.95	0.0097	**0.0485**	protective
DRB4*01:03:01	283	289	19.0%	22.3%	1205	1005	0.82	0.68	0.99	0.03427	0.54832	protective
DRB1*04:02:01	59	79	3.93%	6.04%	1441	1229	0.64	0.44	0.91	0.011040	0.563040	protective
DRB1*04:04:01	24	37	1.60%	2.83%	1476	1271	0.56	0.32	0.96	0.027590	1	protective
DQA1*03:01:01	128	157	8.53%	12.00%	1372	1151	0.68	0.53	0.88	0.002612	**0.070524**	protective
DQB1*03:02:01	117	137	7.80%	10.47%	1383	1171	0.72	0.55	0.94	0.014650	0.571350	protective
DPA1*01:03:01	1151	1047	76.73%	80.05%	349	261	0.82	0.68	0.99	0.03489	0.628020	protective
DPB1*16:01:01	2	10	0.13%	0.76%	1498	1298	0.17	0.02	0.82	0.016570	0.712510	protective

Pc<0.1 are bold numbers.

The HLA alleles frequencies in the PD group (n, 750) compared statistically against those in the Prodrome (n, 57) and SWEDD (n, 60) groups are shown in **Table 4**. There are 11 and 17 allelic differences at *p<0.05* in the PD-Prodrome, and PD-SWEDD comparisons respectively, but only the protective HLA-DQA1*03:03:01 in the PD-Prodrome comparison is significant (*Pc=0.066*) after the Bonferroni correction. Although the expressed HLA-DRA*01:01:01 and -DQA1*03:01:01 are protective alleles in the PD-HC comparison at *Pc<0.1* (**Table 3**), HLA-DQA1*03:01:01 is significant only at the *p<0.05* level and HLA-DRA*01:01:01 is not significant (*p>0.05)* in the SWEDD-HC comparison (**Table 4**). Neither HLA-DRA*01:01:01 nor -DQA1*03:01:01 is significant (*p>0.05)* in the PD-Prodrome comparison (**Table 4**).

**Table 4:** Significant expressed HLA genotypes in Parkinson Disease (PD) compared to (A) prodrome and (B) scans without evidenece of dopamine deficits (SWEDD).

(A) PD, n=750 v Prodrome, n=57												
11 alleles	n		%		PD	PRODROME	OR	95% CI		P < 0.05	P< 0.1	Risk or
Allele name	PD	PRODROME	PD	PRODROME	Other	Other		Lower	Upper	P-value	Pc-value	protective
B*14:02:01	94	1	6.3%	0.9%	1406	113	7.55	1.29	303.82	0.01195	0.87235	risk
DQA1*05:05:01	270	12	18.0%	10.5%	1230	102	1.87	1.004	3.78	0.04105	1	risk
DPB1*04:02:01	182	6	12.1%	5.3%	1318	108	2.48	1.08	7.02	0.02318	0.90402	risk
A*02:01:01	308	35	20.5%	30.7%	1192	79	0.58	0.38	0.91	0.01271	0.59737	protective
A*34:02:01	3	2	0.2%	1.8%	1497	112	0.11	0.01	1.36	0.04295	1	protective
B*51:01:01	57	9	3.8%	7.9%	1443	105	0.46	0.22	1.09	0.04573	1	protective
DRB5*01:02:01	32	6	2.2%	5.3%	1456	108	0.40	0.16	1.18	0.04813	0.81821	protective
DQA1*03:03:01	78	15	5.2%	13.2%	1422	99	0.36	0.20	0.70	0.00244	**0.06583**	protective
DPA1*02:01:08	4	3	0.3%	2.6%	1496	111	0.10	0.02	0.69	0.00974	0.17536	protective
DPB1*14:01:01	20	6	1.3%	5.3%	1480	108	0.24	0.09	0.76	0.00779	0.30369	protective
DPB1*16:01:01	2	2	0.1%	1.8%	1498	112	0.08	0.01	1.04	0.02701	1	protective
(B) PD, n=750 v SWEDD, n=60												
17 alleles	n		%		PD	SWEDD	OR	95% CI		P < 0.05	P< 0.1	Risk or protective
Allele name	PD	SWEDD	PD	SWEDD	Other	Other		Lower	Upper	P-value	Pc-value	
B*14:02:01	94	2	6.3%	1.7%	1406	118	3.94	1.04	33.44	0.04184	1	risk
C*08:02:01	100	2	6.7%	1.7%	1400	118	4.21	1.11	35.70	0.02929	1	risk
DRB absent	239	11	16.1%	9.2%	1249	109	1.90	0.999	3.97	0.04876	0.73140	risk
DQA1*05:05:01	270	13	18.0%	10.8%	1230	107	1.81	0.99	3.56	0.04590	1	risk
DQB1*06:03:01	130	3	8.7%	2.5%	1370	117	3.70	1.21	18.45	0.01442	0.47586	risk
A*02:972	1	2	0.1%	1.7%	1499	118	0.04	0.001	0.77	0.01554	0.73038	protective
B*07:02:01	108	17	7.2%	14.2%	1392	103	0.47	0.27	0.87	0.01138	0.84212	protective
C*07:02:01	130	19	8.7%	15.8%	1370	101	0.50	0.30	0.90	0.01332	0.57276	protective
C*07:19	0	2	0%	1.7%	1500	118	0	0	0.42	0.00545	0.23414	protective
DRB4*01:03:02	10	4	0.7%	3.3%	1478	116	0.20	0.06	0.87	0.01642	0.24630	protective
DRB1*04:04:01	24	7	1.6%	5.8%	1476	113	0.26	0.11	0.74	0.00604	0.27789	protective
DQA1*03:01:01	128	18	8.5%	15.0%	1372	102	0.53	0.31	0.96	0.02923	0.75998	protective
DQB1*03:02:01	117	18	7.8%	15.0%	1383	102	0.48	0.28	0.87	0.00968	0.31941	protective
DQB1*03:03:02	54	9	3.6%	7.5%	1446	111	0.46	0.22	1.09	0.04577	1	protective
DPB1*06:01:01	13	5	0.9%	4.2%	1487	115	0.20	0.07	0.73	0.00803	0.29718	protective
DPB1*10:01:01	20	6	1.3%	5.0%	1480	114	0.26	0.10	0.80	0.00981	0.36286	protective
DPB1*20:01:01	3	3	0.2%	2.5%	1497	117	0.08	0.01	0.59	0.00672	0.24868	protective

The significant differences at *p<0.05* for 43 HLA alleles (25 HLA class I and 38 HLA class II alleles) up to the three-field resolution in two comparisons between HC and the two PD subgroups, Prodrome (A) and SWEDD (B), are shown in **Table 5**. There are 20 different alleles (18 risk and 2 protective) in the Prodrome-HC, and 23 (18 risk and five protective) in the SWEDD-HC comparisons, with five alleles (HLA-A*02:844, -B*14:02:01, -B*40:01:02, -C*03:04:01, -DRB1*04:01:01) overlapping between the two comparisons, (A) and (B). Only three alleles were significant (*Pc<0.1*) after Bonferroni correction, the HLA-DQA1*03:03:01 risk allele in the Prodrome-HC comparison, and the HLA-DRA*01:01:02 and -DRB4*01:03:02 risk alleles in the SWEDD-HC comparison.

**Table 5:** Significantly expressed HLA genotypes in healthy controls (HC) versus (A) prodrome, and (B) scans without evidence of dopamine deficits (SWEDD).

(A) HC, n=654 v PRODROME, n=57												
20 alleles	n		%		PRODROME	HC	OR	95% CI		P < 0.05	P< 0.1	Risk or
Allele name	PRODROME	HC	PRODROME	HC	Other	Other		Lower	Upper	P-value	Pc-value	protective
A*02:01:01	35	250	30.70%	19.11%	79	1058	1.87	1.19	2.90	0.004761	0.20472	risk
A*02:844	2	2	1.75%	0.15%	112	1306	11.61	0.83	161.53	0.034350	1	risk
A*34:02:01	2	0	1.75%	0%	112	1308	Inf	2.17	Inf	0.006375	0.27413	risk
B*40:01:02	7	30	6.14%	2.29%	107	1278	2.78	1.01	6.67	0.024140	1	risk
B*49:01:01	5	19	4.39%	1.45%	109	1289	3.11	0.89	8.83	0.037510	1	risk
B*51:01:01	9	42	7.89%	3.21%	105	1266	2.58	1.07	5.57	0.017110	1	risk
C*03:04:01	9	39	7.89%	2.98%	105	1269	2.79	1.15	6.05	0.011580	0.48636	risk
C*05:01:01	14	85	12.28%	6.50%	100	1223	2.01	1.02	3.73	0.032010	1	risk
DRB5*02:02:01	5	20	4.4%	1.5%	109	1274	2.92	0.84	8.22	0.04560	0.72960	risk
DRB1*04:01:01	10	46	8.77%	3.52%	104	1262	2.64	1.15	5.49	0.011140	0.52358	risk
DRB1*11:02:01	2	2	1.75%	0.15%	112	1306	11.61	0.83	161.53	0.034350	1	risk
DRB1*16:01:01	5	16	4.39%	1.22%	109	1292	3.70	1.04	10.82	0.021780	1	risk
DQA1*01:02:02	6	22	5.26%	1.68%	108	1286	3.24	1.05	8.48	0.020310	0.50775	risk
DQA1*03:03:01	15	71	13.16%	5.43%	99	1237	2.64	1.35	4.86	0.003018	**0.07545**	risk
DQB1*03:19:01	2	1	1.75%	0.08%	112	1307	23.19	1.20	1363.90	0.018120	0.56172	risk
DQB1*05:02:01	6	26	5.26%	1.99%	108	1282	2.74	0.90	6.99	0.037550	1	risk
DPA1*02:01:08	3	4	2.63%	0.31%	111	1304	8.78	1.27	52.62	0.01384	0.22144	risk
DPB1*14:01:01	6	16	5.26%	1.22%	108	1292	4.48	1.41	12.36	0.005985	0.20948	risk
B*14:02:01	1	96	0.88%	7.34%	113	1212	0.11	0.00	0.65	0.005414	0.33567	protective
DQA1*03:01:01	6	157	5.26%	12.00%	108	1151	0.41	0.14	0.94	0.030770	0.76925	protective
(B) HC, n=654 v SWEDD, n=60												
23 alleles	n		%		SWEDD	HC	OR	95% CI		P < 0.05	P< 0.1	Risk or
Allele name	SWEDD	HC	SWEDD	HC	Other	Other		Lower	Upper	P-value	Pc-value	protective
A*02:844	2	2	1.67%	0.15%	118	1306	11.03	0.79	153.62	0.03755	1	risk
A*02:972	2	0	1.67%	0%	118	1308	Inf	2.06	Inf	0.00701	0.30134	risk
B*07:02:01	17	97	14.17%	7.42%	103	1211	2.06	1.11	3.64	0.01342	0.87230	risk
B*40:01:02	8	30	6.67%	2.29%	112	1278	3.04	1.18	6.99	0.01121	0.72865	risk
C*03:04:01	9	39	7.50%	2.98%	111	1269	2.64	1.09	5.72	0.01558	0.65436	risk
C*07:02:01	19	108	15.83%	8.26%	101	1200	2.09	1.16	3.60	0.01067	0.44814	risk
C*07:19	2	0	1.67%	0%	118	1308	Inf	2.06	Inf	0.00701	0.29434	risk
DRA*01:01:02	7	27	5.8%	2.1%	113	1281	2.94	1.06	7.11	0.0197	**0.0983**	risk
DRB4*01:03:02	4	5	3.3%	0.4%	116	1289	8.86	1.73	41.79	0.00445	**0.06671**	risk
DRB5*01:01:01	15	90	12.5%	7.0%	105	1204	1.91	0.99	3.47	0.04241	0.63615	risk
DRB1*04:01:01	9	46	7.50%	3.52%	111	1262	2.22	0.93	4.75	0.04300	1	risk
DQA1*01:02:01	22	140	18.33%	10.70%	98	1168	1.87	1.09	3.11	0.01583	0.39575	risk
DQB1*03:03:02	9	32	7.50%	2.45%	111	1276	3.23	1.32	7.14	0.00539	0.15092	risk
DQB1*06:02:01	18	110	15.00%	8.41%	102	1198	1.92	1.05	3.34	0.02797	0.78316	risk
DPA1*02:01:02	7	28	5.83%	2.14%	113	1280	2.83	1.02	6.82	0.02291	0.32074	risk
DPB1*06:01:01	5	17	4.17%	1.30%	115	1291	3.30	0.93	9.53	0.03173	1	risk
DPB1*10:01:01	6	18	5.00%	1.38%	114	1290	3.77	1.20	10.16	0.01186	0.39138	risk
DPB1*20:01:01	3	5	2.50%	0.38%	117	1303	6.67	1.02	34.75	0.02369	0.78177	risk
B*14:02:01	2	96	1.67%	7.34%	118	1212	0.21	0.03	0.81	0.01330	0.86450	protective
C*08:02:01	2	98	1.67%	7.49%	118	1210	0.21	0.02	0.80	0.01353	0.56826	protective
DRB345 absent	11	239	9.2%	18.5%	109	1055	0.45	0.21	0.85	0.00849	0.12734	protective
DQA1*01:03:01	4	116	3.33%	8.87%	116	1192	0.35	0.09	0.96	0.03756	0.93900	protective
DQB1*06:03:01	3	122	2.50%	9.33%	117	1186	0.25	0.05	0.77	0.00670	0.18752	protective

‘Inf’ is an infinite value due to a zero in HC.

Pc<0.1 are bold numbers.

The statistical analyses of the HLA allele frequency differences at the *p<0.05* level of significance show that the PD, Prodrome and SWEDD subgroups are markedly different from each other within the PPMI cohort (**Tables 3**–**5**). There are 57 significantly *(p<0.05)* different HLA alleles (21 class I and 36 class II) in the five statistical comparisons between the different PD subgroups (**Tables 3**–**5**). Twenty-six (9 class I and 17 class II) of the 57 different alleles are limited to a single subgroup comparison, mainly in the HC-PD (14 of 20 alleles), HC-Prodrome (6 of 20 alleles), HC-SWEDD (4 of 23 alleles) and PD-Prodrome (2 of 11 alleles) comparisons, whereas thirty-one (12 class I and 19 class II) of the 57 alleles overlap between two or more different subgroup comparisons (**Supplementary Table 3**). In addition, there are more protective alleles in the HC group than risk alleles in the PD group at a ratio of 12 to 8 (60%) in the PD-HC comparison (**Table 3**), whereas the Prodrome and SWEDD comparisons with HC have more risk alleles than protective alleles at ratios of 18 to 2 (90%), and 18 to 5 (78%), respectively (**Table 5**). In a statistical comparison between the HC group (n, 654) and the combined PD subgroups (PD, Prodrome and SWEDD, [n, 867]), presented in **Supplementary Table 4**, there are 17 risk and 15 protective HLA alleles (12 class I and 20 class II) with 8 of the 32 significant alleles (p<0.05) present only in this analysis, whereas the other 24 alleles are present in at least one of the other statistical comparisons (**Tables 3**–**5**). In this analysis, the HLA-DRA*01:01:01 protective allele is significant (*P=0.0223*) after a Bonferroni correction.

In summary, only five of the expressed HLA alleles shown in **Tables 3**, **5** are significantly different (*Pc<0.1*) after Bonferroni corrections, HLA-DRA*01:01:01 (HC v PD), -DQA1*03:01:01 (HC v PD), -DQA1*03:03:01 (PD v Prodrome, HC v Prodrome), -DRA*01:01:02 and -DRB4*01:03:02 (SWEDD v HC).

### 3.3 SVA genotyped transcripts and phased-haplotypes within case-control comparisons

The eQTL SVA transcripts expressed at eight MHC loci (*NR_SVA_377, R_SVA_24, R_SVA_25, R_SVA_26, NR_SVA_380, R_SVA_27, R_SVA_85, NR_SVA_381*) that are shown in **Figure 1** had statistically inferred regulatory effects on classical class I and class II gene transcription levels and their different isoforms (44). The MHC SVA genotype frequencies and their influence on classical class I and class II HLA genes and transcripts based on a previous study (44) are shown in **Supplementary Table 5**. The number and percentage frequency of the 64 SVA-phased haplotypes with the eight MHC genotyped SVA as present or absent insertions in the present study are shown in **Supplementary Table 6**.

Significant differences for SVA genotypes were detected at *p<0.05* by the Fisher’s exact test between different subgroups (PD, Prodrome, SWEDD and HC) within the PPMI cohort for only four (*R_SVA_25, NR_SVA_380, R_SVA_85*, and *NR_SVA_381*) of the eight SVAs (**Table 6**). *R_SVA_25* when absent (A) on both chromosomes is a homozygous AA referred to as the *R_SVA_25 AA* genotype. In the PD-SWEDD comparison, the *R_SVA_25 AA* genotype is a PD risk, whereas the *R_SVA_25 PA* genotype is protective. The homozygous presence (*PP*) of the *NR_SVA_381* insertion is a significant risk in the PD-HC and HC-Combination comparisons both at the *p<0.05* and *Pc<0.1* levels, but only at the *p<0.05* level in the HC-Prodrome comparison.

**Table 6:** Significant SVA genotypes transcribed in healthy controls (HC) versus (A) Parkinson Disease (PD), (B) prodrome, (C) Scans Without Evidence of Dopamine Deficits (SWEDD), and (D) combination (A+B+C); and (E) PD versus SWEDD.

(A) HC (n, 654) v PD (n, 750)											
		n		%		OR	95% CI		P<0.05	P<0.1	Risk or
Locus	Genotype	PD	HC	PD	HC		Lower	Upper	P-value	Pc-value	protective
NR_SVA_380	PA	139	148	22.53%	27.82%	0.75	0.57	1.00	0.040530	1.0000	protective
NR_SVA_381	PP	39	10	6.31%	1.88%	3.51	1.70	7.97	0.000190	0.0124	risk
(B) HC (n, 654) v PRODROME (n, 57)											
		n		%		OR	95% CI		P<0.05	P<0.1	Risk or
Locus	Genotype	PRODROME	HC	PRODROME	HC		Lower	Upper	P-value	Pc-value	protective
R_SVA_85	AA	3	5	5.77%	0.97%	6.22	0.94	33.07	0.029170	1.0000	risk
NR_SVA_381	PP	5	10	9.09%	1.88%	5.19	1.34	17.48	0.008732	0.5414	risk
(C) HC (n, 654) v SWEDD (n, 60)											
		n		%		OR	95% CI		P<0.05	P<0.1	Risk or
Locus	Genotype	SWEDD	HC	SWEDD	HC		Lower	Upper	P-value	Pc-value	protective
R_SVA_25	PA	19	82	32.76%	15.50%	2.65	1.37	4.97	0.002712	0.1654	risk
R_SVA_25	AA	39	444	67.24%	83.93%	0.39	0.21	0.76	0.003253	0.1984	protective
NR_SVA_380	PA	9	148	15.25%	27.82%	0.47	0.20	0.99	0.042860	1.0000	protective
NR_SVA_380	AA	50	378	84.75%	71.05%	2.26	1.07	5.36	0.030620	1.0000	risk
(D) HC (n, 654) v Combination (A+B+C) (n, 867)											
		n		%		OR	95% CI		P<0.05	P<0.1	Risk or
Locus	Genotype	All	HC	All	HC		Lower	Upper	P-value	Pc-value	protective
NR_SVA_380	PA	163	148	22.30%	27.82%	0.74	0.57	0.97	0.028960	1.0000	protective
R_SVA_85	AA	19	5	2.70%	0.97%	2.84	1.02	9.78	0.036250	1.0000	risk
NR_SVA_381	PP	47	10	6.42%	1.88%	3.58	1.76	8.02	0.000090	0.0058	risk
(E) PD (n, 750) v SWEDD (n, 60)											
		n		%		OR	95% CI		P<0.05	P<0.1	Risk or
Locus	Genotype	PD	SWEDD	PD	SWEDD		Lower	Upper	P-value	Pc-value	protective
R_SVA_25	AA	510	39	83.1%	67.2%	2.39	1.25	4.43	0.00671	0.43596	risk
R_SVA_25	PA	96	19	15.6%	32.8%	0.38	0.20	0.73	0.00284	0.18441	protective

### 3.4 SVA and HLA phased-haplotypes within case-control comparisons

**Figure 2:**
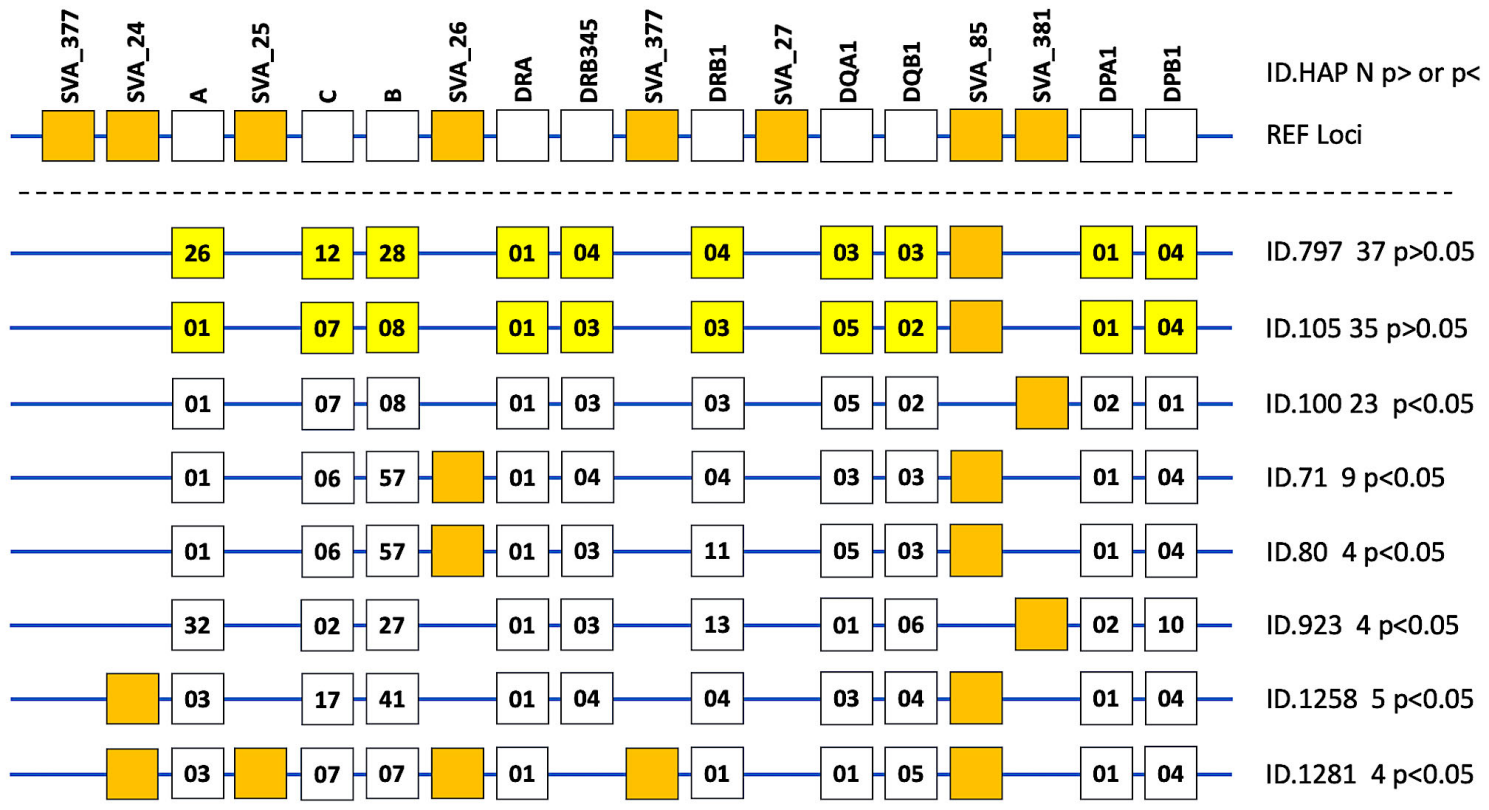
Phased haplotypes of HLA genotypes at 10-loci and the absence or presence of the SVA insertion at 8-loci are presented as line diagrams for eight examples of 1540 different haplotypes listed in **Supplementary Table 7**. The top horizontal line with 18 boxes represents the reference loci (REF loci) of a hypothetical haplotype with the ten labelled HLA genes (open boxes) and all the labelled SVA present at 8 loci. The R and NR designations were omitted from the labelled SVA loci. The next two horizontal lines from the top represent the two most frequent HLA/SVA haplotypes (ID.797 [n, 37] and ID.101 [n, 35], respectively) with ten yellow boxes representing the allelic groups of the HLA genes and the presence of only one SVA (closed orange box) represented by SVA-85. No other SVA was present in these two haplotypes that were not significantly different (p>0.05) between cases and healthy controls. The next six horizontal lines with ID numbers and n values beside them on their right side represent the haplotypes that were significantly different between PD and HC at *p<0.05.* The ten open boxes on each horizontal line represent the allelic groups of the HLA genes labelled on REF loci at the top. The SVA present in one or other of the particular haplotypes are represented by the closed orange boxes. For example, the bottom horizontal line represents the ID.1281 haplotype (n, 4) listed in **Supplementary Table 7** and the orange closed boxes represent the presence of 5 SVA insertions that are SVA_24, SVA_25, SVA_26, SVA_377, and SVA_85. This haplotype does not have a DRB3, 4 or 5 gene, hence there is no open box in the DRB345 column. Also, there is no SVA_377 or SVA_27 insertion in any of these eight phased haplotype examples.

Phased haplotypes and statistical analysis (Fisher’s exact test, *p-*value; and Bonferroni correction, *Pc*-value) of HLA genotypes at 10-loci and SVA genotypes at 8-loci are listed in **Supplementary Table 7**. In this haplotype analysis of 18 loci, we used the genotype data of only 1165 (66%) of the 1521 individuals because of missing or uncertain data at one or more loci in the excluded 365 cases. Of the 1540 different phased haplotypes (66%) from a total of 2330 haplotypes in this analysis, only six are significantly different between PD and HC at *p<0.05* (**Figure 2**). However, none of these *p-*values are significant when corrected by Bonferroni for multiple testing. The two most frequent HLA/SVA haplotypes shown in **Figure 2** and listed in **Supplementary Table 7** are phased-haplotype ID-797 (n, 37, 2.4%) and phased-haplotype ID-105 (n, 35, 2.3%). Moreover, there is only one risk haplotype (ID-100, n, 23, 1.5%) detected by the Fisher’s exact test at *p=0.025* that is more frequent in PD than HC. In this haplotype, the high frequency R_SVA_85 is absent (*A*) and the low frequency SVA_381 is present (*P*). The high-risk HLA haplotype reported by Wissemann et al. (35) with the B*07:02/C*07:02/DRB5*01/DRB1*15:01/DQA1*01:02/DQB1*06:02 alleles is split in our study between 30 different haplotypes by including the *HLA-A, -DPA, -DPB* alleles, and SVA genotypes, and therefore was not significant (**Supplementary Table 8**). In our study, the protective HLA-DRB1*04:04 allele reported by Wissemann et al. (35) is part of the ‘protective’ HLA/SVA phased-haplotype ID-1258 (**Supplementary Table 7**).

The SVAs that associated with the HLA allele groups at >73% are listed in **Table 7**. For example, in the MHC class I region, the low frequency *NR_SVA_377* (6.9%) is associated almost exclusively with the HLA-A*11 allele group, whereas the moderately low frequency *R_SVA_24* (26.5%) is associated mostly with three different HLA-A allelic groups, A*03:01, A*11 and A*30:01. The low-frequency *R_SVA_25* (8.6%) is associated mostly with C*07:02, but also with B*07:02, B*38:02, and B*39:06. On the other hand, the moderately high-frequency *R_SVA_26* (46.2%) is strongly associated with at least 11 different *HLA-B* allelic groups and 6 different *HLA-C* allelic groups. *R_SVA_26* is associated at varying low levels (0-25%) with at least 19 different *HLA-B* allelic groups (B*8, B*37, B*38, B*39, B*41, B*42, B*44, B*45, B*46, B*47, B*48, B*49, B*50, B*52, B*53, B*55, B*56, B*58, B*73). In addition, there are various low and high-percentage associations within the same *HLA-B* allelic groups. For example, low percentage associations occur with B*07:05 (but not B*07:02, B*07:04, B*07:06), B*14:02 (but not B*14:01), B*27:02, B*27:05 (but not B*27:07), and B*35:01 (but not B*35:02, B*35:03, B*35:08). While *R_SVA_26* has high percentage associations with HLA-C*03:04 (93.7%) and C*07:02 (82.8%), it has little or no association with HLA-C*03:02 and -C*07:01, respectively.

**Table 7:** SVA insertions and HLA allele associations (>70%).

SVA	HLA allele	% Association	Allele Fraction
NR_SVA _377	A*11:01:01	93.2	68 of 73
(n, 108, 6.9%)	A*11:303	88.1	37 of 42
R_SVA_24	A*03:01:01	100	193 of 193
(n, 418, 26.5%)	A*11:01:01	98.6	72 of 73
	A*11:303	100	42 of 42
	A*30:01:01	100	61 of 61
R_SVA_25	C*07:02:01	88.2	134 of 152
(n, 135, 8.6%)	C*07:02:80	100	1 of 1
	B*07:02:01	91.5	107 of 117
	B*07:02:45	100	1 of 1
	B*38:02:01	100	1 of 1
	B*39:06:02	100	12 of 12
R_SVA_26	B*07:02:01	98.3	123 of 126
(n, 727, 46.2%)	B*13:02:01	100	38 of 38
	B*14:01:01	83.3	10 of 12
	B*18:01:01	95.8	69 of 72
	B*27:07:01	100	5 of 5
	B*35	89.6	95 of 106
	B*40	96.8	90 of 93
	B*51:01:01	87.8	72 of 82
	B*52:01:01	93.3	28 of 30
	B*57	100	47 of 47
	B*81	100	3 of 3
	C*03:04	93.7	74 of 79
	C*07:02	82.8	111 of 134
	C*12:02	100	29 of 29
	C*14	93.3	14 of 15
	C*15	86.6	58 of 67
	C*18	100	4 of 4
NR_SVA _380	DRB1*01:01:01	93.8	181 of 194
(n, 206/1576, 13.1%)	DRB1*10:01:01	100	20 of 20
R_SVA_27	DRB1*15	100	145 of 145
(n, 188/1576, 11.9%)	DRB1*16	100	43 of 43
	DQA1*01:01:01	0.7	1 of 135
	DQA1*01:02:01	58.9	113 of 192
	DQA1*01:02:02	95.5	42 of 44
	DQA1*01:02:04	100	2 of 2
	DQA1*01:03:01	24.4	29 of 119
	DQB1*05:01:24	100	1 of 1
	DQB1*05:02:01	86.5	45 of 52
	DQB1*05:03:01	1.7	1 of 58
	DQB1*06:01:01	73.7	28 of 38
	DQB1*06:01:03	100	2 of 2
	DQB1*06:02:01	75.7	103 of 136
	DQB1*06:03:01	5.2	7 of 135
R_SVA_85	DPA1*01	99.9	1262 of 1263
(n, 1303/1586, 82.2%)	DPA1*03	100	5 of 5
	DPA1*04	100	1 of 1
	DPB1*02	96.5	278 of 288
	DPB1*03	100	101 of 101
	DRB1*04	97	731 of 754
	DPB1*06	100	26 of 26
	DPB1*15	100	20 of 20
	DPB1*16	100	16 of 16
	DPB1*18	100	4 of 4
	DPB1*20	100	10 of 10
	DPB1*23	100	10 of 10
	DPB1*34	100	3 of 3
	DPB1*104	100	50 of 50
	DPB1*105	100	5 of 5
	DPB1*124	100	5 of 5
NR_SVA _381	DPA1*02:01:01	98.1	205 of 209
(n, 313/1586, 19.8%)	DPA1*02:01:02	100	33 of 33
	DPA1*02:01:04	100	3 of 3
	DPA1*02:01:08	100	4 of 4
	DPA1*02:02:02	97.5	39 of 40
	DPA1*02:06	80	8 of 10
	DPA1*02:07:01	100	15 of 15
	DPA1*02:12:01	100	1 of 1
	DPA1*02:26:01	100	2 of 2
	DPA1*04:01:01	100	1 of 1
	DPA1*01:58	100	2 of 2
	DPB1*01	100	55 of 55
	DPB1*05	94.7	36 of 38
	DPB1*09	100	8 of 8
	DPB1*10	100	27 of 27
	DPB1*11	100	13 of 13
	DPB1*13	85.1	40 of 47
	DPB1*14	95.8	23 of 24
	DPB1*17	97.7	43 of 44
	DPB1*19	100	9 of 9

In the MHC class II region, *R_SVA_27* (11.9%) is associated with DRB1*15 and DRB1*16 allelic groups at 100% each. Although *R_SVA_27* is not significant (*p>0.05*) in the PPMI cohort subgroup comparisons, DRB1*15:01 and DRB1*16:01 are significant at *p<0.05 i*n the combination-HC, and Podromal-HC comparisons, respectively. **Supplementary Table 9** shows the number of SVA_27/DRB1*15 or DRB1*16/DQA1 haplotypes in the PPMI cohort, including DRB1*15:01/DQA1*01:02, which is associated with protection from T1D (54) and susceptibility for multiple sclerosis (55). Of the 194 DRB1*15:01/SVA_27 haplotypes in PPMI, 178 (91.8%) are linked to DQA1*01:02/DQB1*06:02. Of the 50 DRB1*16/SVA_27 haplotypes, 39 (78%) are linked to DQA1*01:02:02/DQB1*05:02:01.

The high frequency *R_SVA_85* insertion (82.2%) is associated strongly with three *DPA1* allele groups at 99.9% or 100% and with at least 13 *DPB1* allelic groups at >96%. The relatively low frequency *NR_SVA_381* (19.8%) that is significantly more prevalent in PD than healthy controls (**Table 6**) is strongly associated with the HLA-DPA1*02 and –DPA1*04 allele lineages, and with at least 9 *HLA-DPB1* allele lineages (**Table 7**). Of these *HLA-DPB1* allele groups, DPB1*01, DPB1*10, and DPB1*14 appear to imply a disease risk based on *p<0.05* and high *OR* values >1 (**Tables 3**–**5**).

The HLA alleles DRB1*01, DRB1*04, DRB1*11, DRB1*15, DRB1*16, DQA1*01, DQA1*03, -DQB1*03, -DQB1*05, -DQB1*06 (**Tables 3**–**5**) and *NR_SVA_380* (**Table 6**) are significant (*p<0.05*) in the PPMI cohort subgroup comparisons. In this regard, on the basis of the phase-haplotype inferences, we constructed twelve haplotypes of *NR_SVA_380*, and *HLA-DRB1, -DQA1* and -*DQB1* alleles to estimate their frequency and overall pattern of distribution (**Table 8**). There are 206 *NR_SVA_380* insertions associated with 395 *DRB1/DQA1/DQB1* haplotypes at 52.2%.

**Table 8:** Frequencies of twelve 3-loci HLA-DRB1/DQA1/DQB1 haplotypes with NR_SVA _380 insertions.

1	SVA_380/DRB1*01:01:01/DQA1*01:01:01/DQB1*05:01:01	93.6%	117 of 125
2	SVA_380/DRB1*01:01:02/DQA1*01:01:02/DQB1*05:01:01	94.8%	54 of 57
3	SVA_380/DRB1*10:01:01/DQA1*01:05:01/DQB1*05:01:01	100%	19 of 19
4	SVA_380/DRB1*01:03:01/DQA1*01:01:01/DQB1*05:01:01	80%	4 of 5
5	SVA_380/DRB1*01:03:01/DQA1*05:05:01/DQB1*03:01:01	75%	3 of 4
6	SVA_380/DRB1*08:01:01/DQA1*04:01:01/DQB1*04:02:01	10%	3 of 30
7	SVA_380/DRB1*10:01:01/DQA1*01:05:01/DQB1*06:03:01	100%	1 of 1
8	SVA_380/DRB1*01:01:01/DQA1*01:01:01/DQB1*05:23:01	100%	1 of 1
9	SVA_380/DRB1*01:01:01/DQA1*01:01:04/DQB1*05:01:01	100%	1 of 1
10	SVA_380/DRB1*01:01:01/DQA1*01:02:01/DQB1*05:04	100%	1 of 1
11	SVA_380/DRB1*08:01:01/DQA1*04:02/DQB1*04:02:01	12.50%	1 of 8
12	SVA_380/DRB1*07:01:01/DQA1*02:01:01/DQB1*02:02:01	0.7%	1 of 143
**Total:** 206 SVA_380 insertions associated with 395 DRB1/DQA1/DQB1 haplotypes at 52.2%			

**Figure 3:**
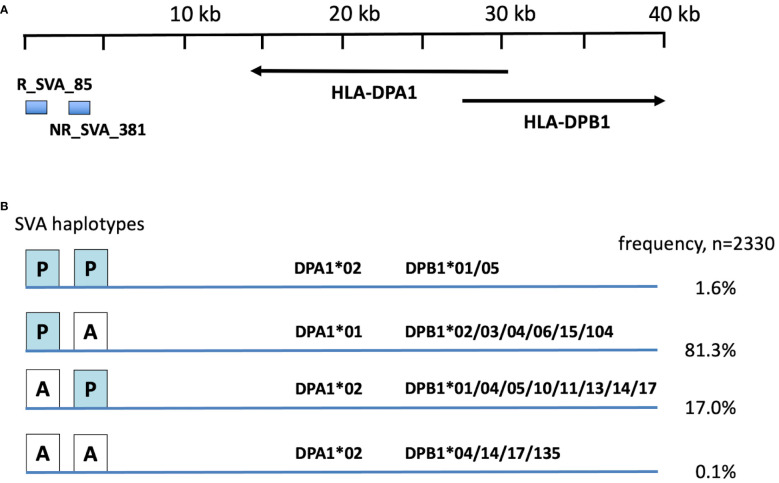
Frequency of R_SVA_85, SVA_381, DPA1, DPB1 haplotypes in PPMI cohort based on phased haplotypes in **Supplementary Table 7**. **(A)** relative position of SVA RIPs and HLA-DP genes at the centromeric end of the MHC class II gene cluster (**Figure 1**). Horizontal arrows show the 5’ prime to 3’ direction of the DPA1 and DPB1 gene coding. **(B)** Four main R_SVA_85, SVA_381 phased haplotype structures, PP, PA, AP and AA, of the 2330 haplotypes listing their percentage frequency association with the DPA1 and DPB1 haplotype allelic lineages.

Because *HLA-DPA1*and *-DPB1* (**Tables 3**–**5**) and *R_SVA_85* and *NR_SVA_381* (**Table 6**) are significant in the PPMI cohort subgroup comparisons, we constructed fifty-five phased haplotypes of the *R_SVA_85, SVA_381* genotypes, and *HLA-DPA1* and *HLA-DPB1* allele lineages to estimate their frequency and overall distribution (**Supplementary Table 10**). **Figure 3** shows that thirty-eight (1.6%) of the 2330 haplotypes have both SVA insertions (PP); 1893 (81.3%) have the R_SVA_85 insertion, but not NR_SVA_381 (PA); 396 (17%) have the NR_SVA_381 insertion, but not the R_SVA_85 (AP); and three (0.1%) have no R_SVA_85 and no NR_SVA_381 (AA). **Supplementary Table 10** reveals that the three most frequent of the *R_SVA_85* insertion/DP haplotypes are R_SVA_85/DPA1*01/DPB1*04 (50.3%), R_SVA_85/DPA1*01/DPB1*02 (15.9%), and R_SVA_85/DPA1*01/DPB1*03 (5.9%). The most frequent *NR_SVA_381* insertion/DP haplotype is NR_SVA_381/DPA1*02/DPB1*01 (3.7%).

### 3.5 Modulation of *HLA-DPA1* and *-DPB1* by two MHC SVA RIPs in the PPMI cohort

**Figure 4:**
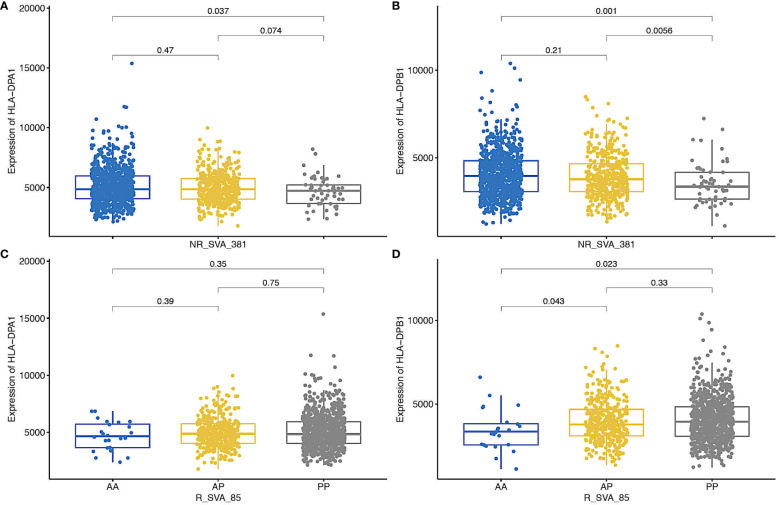
Box plots of regulation of the expression of HLA-DPA1 and HLA-DPB1 transcripts by NR_SVA_381 genotypes [**(A, B)**, respectively], and HLA-DPA1 and HLA-DPB1 transcripts by NR_SVA_85 genotypes (**C, D**, respectively) in PPMI cohort. The genotypes are absent-absent (AA), absent-present (AP), and present-present (PP). The number of genotypes (n) for NR_SVA_381 in each **(A, B)** are 805 for AA, 404 for AP, and 57 for PP in 1266 individuals. The number of genotypes for R_SVA_85 in each **(C, D)** are 24 for AA, 371 for AP and 826 for PP for 1221 individuals, data were not available for 45 individuals. The statistical p-values are shown above the box plots on horizontal lines between the genotypes.

**Figure 4** shows box plots of the possible effects of *R_SVA_85* and *NR_SVA_381* on the expression of *HLA-DPA1* and *-DPB1* transcription. Homozygous *R_SVA_85* insertion (*PP*) significantly increases (*p=0.023*) the transcription of *HLA-DPB1*, but has no significant effect (*p=0.35*) on the transcription of *HLA-DPA1*. The absence of *R_SVA_85* appears to be a risk factor for the Prodrome cohort (**Table 6**). In contrast, homologous *NR_SVA_381* insertion (*PP*) (**Table 6**) significantly decreases the transcription levels of *HLA-DPA1* (*p=0.037*) and *HLA-DPB1* (*p=0.001*) in the PPMI cohort, and its presence as a homozygous genotype (*PP*) is a risk factor (*Pc=0.012*) for PD (**Table 6A**; **Figure 4**).

## 4 Discussion

The regulatory effects of eight transcribed SVA RIPs on the differential co-expression of 71 genes within the MHC genomic region including all the classical class I and class II genes of a PPMI cohort were previously identified by eQTL statistical analysis (41, 43, 44). In this study, the same PPMI RNAseq database was reused to genotype the transcripts encoded by classical class I and class II HLA genes in order to determine their frequency and estimate their haplotypic associations with each other and with the eight regulatory MHC SVAs. The *arcasHLA* software tool (50) was used to impute the genotypes of the transcripts expressed by the class I and class II HLA genes to at least the three-field resolution that included the ancestral allele group, the protein type and the synonymous changes in the coding regions. In a recent comparison of the seven best of 22 genotyping computation tools, *arcasHLA* was the fastest and among the top three most accurate (99.1% for MHC-I and 98.1% for MHC-II) for genotyping Caucasian American RNA data (56). The PPMI cohort HLA allele frequency and haplotype data confirmed that our cohort was mostly (>95%) Caucasian American or Caucasian European as expected (33, 48). Consequently, we have accepted the high accuracy and reliability of the *arcasHLA* imputations without resorting to the use of other genotyping tools.

Significant differences were detected at *p<0.05* by the Fisher’s exact test for 21 HLA class I alleles and 36 HLA class II alleles transcribed by 10 HLA genes that were different up to the three-field resolution within four subgroups (PD, Prodrome, SWEDD and HC) of the PPMI cohort when not corrected by multiple testing (**Tables 3**–**5**). Only five alleles, all from the HLA class II region, were significant after the Bonferroni correction; the expressed protective alleles HLA-DRA*01:01:01 and -DQA1*03:01:01 within PD, the risk allele HLA-DQA1*03:03:01 within the Prodrome cohort and the HLA-DRA*01:01:02 and -DRB4*01:03:02 risk alleles in the SWEDD group. Although HLA-DQA1*03:01:01 differs from -DQA1*03:03:01 by a single nucleotide substitution in exon 3 at codon 160 (c548.C<A), the OR calculations showed that the former was a protective allele and the later a risk allele. HLA-DQA1*03:01:01 was more prevalent at 10.2% than -DQA1*03:03 at 5.7% in the PPMI cohort (**Supplementary Table 2**). The 52 alleles that did not survive the Bonferroni statistical challenge, but had significant differences *p<0.05* between the different cases and controls by the Fisher exact test were placed within a statistically marginal zone of ‘possible’ rather than ‘strong’ or ‘definite’ risk or protective effects. This lower level of statistical significance might have been confounded by various factors such as lack of statistical power due to insufficient sample numbers, unreliable disease and aetiological factors, or various comorbidities and other issues not accounted for in our analysis. However, many of the 57 possible protective or susceptibility HLA alleles (*p<0.05* or *Pc<0.1*) were reported previously by others to be statistically significant in PD and various autoimmune disease studies. For example, we confirmed the results of previous studies that HLA-DRA*01, -DRB4*01:03, -DRB5*01, -DQB1*05 and -C*07:01:01 are predisposing alleles and that HLA-DRB1*04:04, -DQA1*03:01, -DQA1*03:02, and -DQB1*03:02, are protective in PD (34–37, 57). The HLA-A*31:01:02, and -B*40:02:01 possible risk alleles in our PD cohort were not reported previously, although they were associated with the development of acquired aplastic anemia (58). Also, we found that the HLA-DRB1*11:02:01 was a minor possible risk allele in the PD, Prodrome and combined cohorts, but not in the SWEDD cohort. This might be the first report to associate HLA-DRB1*11:02:01 as a possible risk allele in PD, although it has been associated with systemic juvenile idiopathic arthritis (59), Graves’ disease (60) and MS (57).

In our study, there was an overall greater number of possible protective alleles than risk alleles at a ratio of 12 to 8 (60%) in the PD group compared to healthy controls, whereas the Prodrome and SWEDD comparisons with HC had more possible risk alleles than protective alleles at ratios of 18 to 2 (90%), and 18 to 5 (78%), respectively. This greater ratio of HLA risk to protective alleles in Prodrome and SWEDD compared to HC might in part explain the gradual or variable progression to PD. Idiopathic, spasmodic and prodromal PD groups have a mixed population of different HLA haplotypes and HLA alleles that carry and present various peptides and antigens to T lymphocytes, which in turn are activated to regulate a diversity of immune responses including inappropriate and harmful autoimmune responses that can cause extensive tissue damage. In this study, we could not discern easily, which are the possible high risk HLA haplotypes that lead to a faster rate of disease onset, and which are protective or low risk HLA alleles that slow down the disease rate. Wissemann et al. (35) on the basis of their study suggested that the 7.1 ancestral haplotype (AH) that consists of the linked HLA alleles B*07:02/C*07:02/DRB5*01/DRB1*15:01/DQA1*01:02/DQB1*06:02 is a PD high risk haplotype and that the C*03:04, DRB1*04:04 and DQA1*03:01 alleles are part of low-risk haplotypes. We found that five of the alleles in the possible high risk 7.1AH were present in the SWEDD-HC comparison, but not in the PD-HC or Prodrome-HC comparisons. DRB1*15:01 was a possible risk allele only in the Combination (all subgroups)-HC comparison. Also, these high-risk alleles were present in the SWEDD group at a frequency of between 14.2% and 18.3% relative to a frequency between 7% and 10.7% in the HC group. The low-risk alleles DRB1*04:04 and DQA1*03:01 were distributed as protective alleles in our PD-HC comparison. In contrast, C*03:04 was a risk allele in the Prodrome-HC, and the SWEDD-HC comparisons. Furthermore, none of the HLA alleles of the frequent Caucasian 8.1AH haplotype: HLA-A*0101/C*0701/B*0801/DRB1*0301/DQA1*0501/DQB1*0201, except for C*0701, were significant in our study groups.

The statistical results for the HLA-DRB1*04 alleles in previous studies of PD suggested both susceptibility (36) and protective associations (34, 35, 37–39). Our results revealed that the HLA-DRB1*04 alleles were significant statistically at the ‘possible’ level (uncorrected Fisher’s exact test, *p<0.05*) with HLA-DRB1*04:02 and -DRB1*04:04 protective in PD and PPMI (**Table 3**), whereas HLA-DRB1*04:01 was a possible risk allele in the Prodrome and SWEDD groups, and the PPMI cohort (**Table 5**). This difference between the PD, Prodrome, and SWEDD groups might reflect that neither the Prodrome, nor SWEDD groups were an established PD with as yet degenerated dopaminergic neurons and large aggregates of alpha-synuclein or tau proteins (48). A recent study by Mignon et al. (61), reported in a preprint, suggested that HLA-DRB1*04 alleles strongly bound to an epitope sequence of tau in neurofibrillary tangles and mediated an adaptive immune response against tau to decrease PD risk. This protective effect of the HLA-DRB1*04 antigen was intermediary with HLA-DRB1*04:01 and HLA-DRB1*04:03, and absent for HLA-DRB1*04:05 (61), which might explain in part the differentiated statistical results that we obtained for the HLA-DRB1*04 alleles in the PPMI cohort (**Tables 3**–**5**). In contrast, HLA-DRB1*04:01 might transport alpha-synuclein to the cell surface of T-cells (62) to become a risk factor for individuals in the Prodrome and SWEDD groups (**Table 5**). According to Hollenbach et al. (38), HLA-DRB1*04:01 is part of the protective Caucasian haplotype DRB1*04:01/DQA1*03:01/DQB1*03:02 and that, along with DRB1*01:01, has the ‘shared epitope’ (SE) with the amino acid motif Q/RK/RRAA at positions 70–74 in combination with valine at position 11 (11-V) that are highly protective in PD. In our study, HLA-DRB1*01:02 was a possible protective allele within the combined cohort-healthy controls comparison.

Four possible HLA risk alleles, HLA-DRB5*01:01, -DRB1*04:01, -DRB1*15:01, and -DQB1*03:01, are of particular interest because they have been associated with alpha-synuclein specific T cell reactivity in patients with PD (23–25). In this regard, Ozono et al. (62) showed experimentally that HLA class II molecules with the DRB5*01:01 allele captured and transported conformationally abnormal alpha-synuclein extracellularly, whereas HLA-DRB1*04:01 transported normal alpha-synuclein to the cell surface to present to circulating CD4-positive T cells, but did not translocate structurally abnormal alpha-synuclein. Moreover, alpha-synuclein32-46 peptide immunisation of mice that expressed HLA-DRB1*15:01 triggered intestinal inflammation, enteric neurodegeneration, constipation, and weight loss (22), suggesting a critical role for alpha-synuclein autoimmunity in HLA-DRB1*15:01 carriers in the combined PPMI cohort (**Supplementary Table 4**). The findings by Garretti et al. (22) are consistent with the hypothesis that alpha-synuclein-mediated pathology can originate in the enteric neural system and proceed into the brain via the vagus nerve (12, 52). In this context, Braak’s hypothesis (12) connects the onset of PD to the alleles HLA-DRB1*15:01, -DRB1*04:02:01, -DQA1*03, and -DQB1*03:02:01 that are associated with Crohn’s disease, colitis or celiac disease (63–65), and that we found were significant (*p<0.05*) in the PPMI cohort (**Tables 3**–**5**; **Supplementary Table 4**). The question remains whether the CD4+ T lymphocytes that recognise and interact with the presented HLA class II bound alpha-synuclein antigens might in turn trigger cytotoxic CD8+ T lymphocytes and antibody producing B-lymphocytes to attack and destroy neurons that display HLA-bound alpha-synuclein antigen at the cell surface in the peripheral and central nervous systems. A dysfunctional blood brain barrier in PD patients can lead to increased levels of alpha-synuclein, autoantibodies against alpha-synuclein, and infiltrating T cells in the CSF and plasma (66). Consequently, more information is required about what subgroups of autoreactive T and B lymphocytes and other self-antigens beside alpha-synuclein might be generated by the adaptive immune system in PD pathogenesis.

Eight SVA eQTLs expressed within the MHC region were inferred to differentially modulate the transcription levels of classical class I and class II HLA genes within the PPMI cohort (43, 44). In the present study, four of the eight regulatory SVA-RIPs, *R_SVA_25, NR_SVA_380, R_SVA_85* and *NR_SVA_381*, are significant (*p<0.05)* by the Fisher’s exact test within the different PPMI subgroups, but only the *SVA_381 PP* genotype is significant (*Pc<0.1*) after Bonferroni corrections for multiple testing (**Table 6**). *SVA_381 PP* is a significant (p<0.05) risk in the PD-HC, Prodrome-HC and combination-HC comparisons, but not significant (p>0.05) in the SWEDD-HC comparison. This result might be related to the observation that the homologous *NR_SVA_381* insertion (*PP*) is associated significantly with a decrease in the transcription levels of *HLA-DPA1* (*p=0.037*) and *HLA-DPB1* (*p=0.001*) in the PPMI cohort (**Figure 4**). The suppressed transcription rate might result in a reduced level of HLA-DPA1 and -DPB1 antigen presentation to the circulating CD4+ helper cells. Previously, *NR_SVA_381* was inferred to modulate only the allelic expression of the *HLA-DPA1*, -*DPB1* and *-B* genes, whereas *R_SVA_85* only modulates the *HLA-DPA1* and *-DPB1* genes (44). Thus, *R_SVA_85* and *NR_SVA_381* might have opposing regulatory effects on the *HLA-DPA1*, and *-DPB1* gene expression that together could have a small, but significant effect in some PD and Prodrome cases (**Figures 3**, **4**).

The total absence of *R_SVA_85* (genotype *AA*) is a minor risk factor for the Prodrome cohort (**Table 6**), which suggests that its presence (genotype *PP*) might be protective. A possible protective role is supported by its presence in four significant protective haplotypes (**Figure 2**). Although *R_SVA_85* significantly increased (*p=0.023*) the transcription of *HLA-DPB1*, its presence (*PP*) had no significant statistical effect (*p>0.05*) on the levels of *HLA-DPA1* transcription (**Figure 4**). Therefore, the protective effect of the presence of *R_SVA_85* in PD or in the Prodrome cohort might be diluted out in a statistical analysis because of its overall high frequency (82.9%) in the PPMI cohort and strong association with many different *HLA-DPA1* alleles (**Table 7**; **Figure 3**). Although the *R_SVA_85* and *NR_SVA_381* loci are separated from each other by 1.7 kb in an intergenic region between the *HLA-DOA* and *HLA-DPA1* genes (**Figure 1**), they are together only at a low frequency of 1.6% (**Figure 3**). The effects of *R_SVA_85* and *NR_SVA_381* on the gene expression of *HLA-DPA1* and -*DPB1* genes (**Figure 4**), either separately or together, is of interest also for unrelated hematopoietic cell transplantation because the level of expression of HLA-DP in the recipient is an important prognostic indicator of donor-anti-host recognition and for evaluating the risk of graft-versus-host disease (67, 68).

Of the two other SVA-RIPs that had minor significance (p<0.05), *R_SVA_25* modulates the transcription of all the HLA class I genes and *HLA-DRB5, -DRB1 and -DQB1*, whereas *NR_SVA_380* modulates the transcription of the *HLA-C, HLA-DRB1* and the two *HLA-DQ* genes (44)*. R_SVA_25* is inserted 23.4 kb telomeric of *HLA-C*, occurs at low frequency (8.6%), but upregulates the expression of 88.2% of the C*07:02:01 risk alleles and 92% of the B*07:02:01 risk alleles (**Table 7**) in the SWEDD-HC comparison (**Table 4**). *SVA_380 AA* is a minor risk genotype in the SWEDD-HC comparison, while *SVA_380 PA* is protective in the PD-HC comparison as well as in the SWEDD-HC comparison (**Table 6**). *NR_SVA_380*, inserted between *DRB5* and *DRB6*, up-regulates 93.8% of DRB1*01:01:01 and 100% of DRB1*10:01:01 neutral alleles (**Table 7**).

Our previous study was unable to discern whether *SVA_24, SVA_380* or *SVA_27* modulated the expression of *HLA-DQA1* protective or risk alleles (44). The present study revealed that of the 110 HLA-DQA1*03:03:01 risk alleles in the PPMI cohort, 31 (28.2%) were associated with *SVA_24*, none with *SVA_380*, and none with *SVA_27*. Similarly, of 147 DQA1*03:01:01 protective alleles, 35 (23.7%) were associated with *SVA_24*, none with *SVA_380*, and none with *SVA_27*. Although *SVA_24* is located near *HLA-A* within the alpha block of the MHC class I region and located 2.7 Mb from the *HLA-DQA1* locus, it appears to regulate the expression levels of the two most statistically significant (Pc<0.1) HLA class II alleles, HLA-DQA1*03:01:01 and -DQA1*03:03:01, detected in our study (**Tables 3**, **5**, respectively). If this is the case, then the statistical significance of *SVA_24* transcription and regulation of HLA alleles in PD was not detected probably because it is associated more strongly with transcription modulation of the *HLA-A3, -A11* and *-A30* neutral alleles (**Table 7**). None of the other transcribed SVA RIPs were associated with the significant HLA-DQA1*03 alleles.

SVAs have been inserted and translocated at different times of human evolutionary history (69). Consequently, the MHC SVA are associated more strongly with some HLA allele groups than others and could be used as evolutionary and disease markers (45). For example, *R_SVA_27*, although present in 11.9% of the PPMI cohort, is associated with all of the HLA-DRB1*15 and -DRB1*16 alleles, but with none of the other 11 *HLA-DRB1* allelic lineages (**Table 7**). We hypothesize that *R_SVA_27* was inserted originally into the MHC class II region in a location between *HLA-DRB1* and *HLA-DQA1* in an ancestor with either HLA-DRB1*15 or -DRB1*16 or a heterozygote individual who had both alleles. *R_SVA_27* is associated strongly also with some of the HLA-DQB1*05 or -DQB1*06 lineage alleles (**Table 7**). Therefore, *R_SVA_27* is an unique autoimmune disease marker because its linkage to the DRB1*15:01/DQA1*01:02/DQB1*06:02 haplotype has been associated with multiple sclerosis (38, 70–72), and the R_SVA_27/DRB1*16/DQB*05 haplotype was associated with other autoimmune diseases (73, 74). In the PPMI cohort, *R_SVA_27* is associated strongly with HLA-DQA1*01:02 (96%), but weakly or not at all with the other -DQA1*01 field-three alleles such as HLA-DQA1*01:01:01 (0.7%), -DQA1*01:02:01 (58.9%), or -DQA1*01:03:01 (24.4%). These lower percentage associations suggest that HLA-DQA1*01:01:01 was already fully established in the population before *R_SVA_27* was inserted, and that HLA-DQA1*01:02:02 emerged probably at a time proximate to the *R_SVA_27* insertion event.

This study confirms and extends our previous reports that transcribed SVA elements inserted within the MHC genomic region can modulate certain HLA genes at the transcription level (41, 43, 44), and therefore, might regulate the expression of particular HLA risk and protective alleles, which in turn influence the onset and progression of PD via the immune response. For example, the upregulated or downregulated HLA transcription levels modulated by SVA transcripts could change the levels of foreign or autoreactive self-peptide presentation to CD4+ T helper lymphocytes or cytotoxic CD8+ T lymphocytes and influence the onset, development or progression of PD. In this regard, the SVA and HLA PD risk variants are likely additive causes with a complicated polygenic structure. Stronger statistical and molecular significance might be found in future studies with better stratification and compartmentalisation of the PD co-morbidities associated with autoimmune diseases and other age-related neurological diseases. The coordination of the adaptive and innate immunity by the HLA system in PD is highly complex and still poorly understood (3, 8, 66). The infiltration of peripheral CD4+ and CD8+ lymphocytes and monocytes into the brain across a dysfunctional blood brain barrier however suggests that the adaptive immune system contributes to neurodegeneration at different stages of PD pathogenesis (10, 14, 26, 66). While we limited our analysis to eight SVA within the MHC genomic region, it is noteworthy that there are many SVAs in other genomic regions that are strongly linked to PD (42, 43), including the SVA insertion within the *TAF1* gene that is associated with X-linked dystonia parkinsonism (75).

In conclusion, our study of the expressed *SVA* and *HLA* genes in circulating white blood cells confirms that the MHC genomic region has an important role in the coordinated regulation of immune responses possibly associated with the long-term onset and progression of PD, the mechanisms of which yet have to be elucidated. MHC SVA RIPs, by down or up regulating the antigen presenting HLA alleles at the proteomic level, might change the amount of risk or protective antigens presented to the CD4+ or CD8+T helper lymphocytes. Thus, co-expression of regulatory SVA RIPs and HLA class I and class II alleles adds another layer of biomolecular complication to the understanding of immune responses associated with PD.

## Data availability statement

The original contributions presented in the study are included in the article/**Supplementary Material**. Further inquiries can be directed to the corresponding author.

## Ethics statement

The studies involving humans were approved by University of Western Australia Human research ethics office. The studies were conducted in accordance with the local legislation and institutional requirements. The participants provided their written informed consent to participate in this study.

## Author contributions

JK: Writing – original draft, Methodology, Writing – review & editing, Formal analysis, Conceptualization. SS: Writing – review & editing, Methodology. TS: Writing – review & editing. AP: Data curation, Writing – review & editing. SK: Project administration, Formal analysis, Conceptualization, Writing – review & editing.
